# Bioinformatic analysis and experimental validation of cuproptosis-related LncRNA as a novel biomarker for prognosis and immunotherapy of oral squamous cell carcinoma

**DOI:** 10.1186/s41065-024-00311-5

**Published:** 2024-02-27

**Authors:** Shuang Liang, Lanting Ji, Zhenyuan Yu, YaHsin Cheng, Ruifang Gao, Wenpeng Yan, Fang Zhang

**Affiliations:** 1 Department of Oral Medicine, Shanxi Province Key Laboratory of Oral Diseases Prevention and New Materials, Shanxi Medical University School and Hospital of Stomatology, Taiyuan, 030001 China; 2 Department of Physiology, School of Medicine, China Medical University, Taichung, 40402 Taiwan

**Keywords:** Cuproptosis, lncRNA, Oral squamous cell carcinoma, Prognostic model, Biomarkers, Immune response, Drug sensitivity

## Abstract

**Background:**

The novel form of regulatory cell death, cuproptosis, is characterized by proteotoxicity, which ultimately leads to cell death. Its targeting has emerged as a promising therapeutic approach for oral squamous cell carcinoma (OSCC). Long noncoding RNAs (lncRNAs) participate in epigenetic regulation and have been linked to the progression, prognosis, and treatment of OSCC. Thus, this study aimed to identify new cuproptosis-related lncRNAs (CRLs), establish predictive models for clinical prognosis, immune response, and drug sensitivity, and provide novel insights into immune escape and tumor drug resistance.

**Methods:**

The present study screened eight CRLs (THAP9-AS1, STARD4-AS1, WDFY3-AS2, LINC00847, CDKN2A-DT, AL132800.1, GCC2-AS1, AC005746.1) using Lasso Cox regression analysis to develop an eight-CRL prognostic model. Patients were categorized into high- and low-risk groups using risk scores. To evaluate the predictive ability of the model, Kaplan-Meier analysis, ROC curves, and nomograms were employed. Furthermore, the study investigated the differences in immune function and anticancer drug sensitivity between the high- and low-risk groups. To validate the expression of CRLs in the model, OSCC cell lines were subjected to quantitative real-time fluorescence PCR (qRT-PCR).

**Results:**

The results of the study showed that the high-risk group had a shorter overall survival (OS) time in OSCC patients. Cox regression analysis demonstrated that the high-risk score was an independent risk factor for a poor prognosis. The validity of the model was confirmed using ROC curve analysis, and a nomogram was developed to predict the prognosis of OSCC patients. Furthermore, patients in the high-risk group with high TMB had a poorer prognosis. Patients in the low-risk group responded better to immunotherapy than those in the high-risk group. Additionally, the risk scores were significantly associated with drug sensitivity in OSCC patients. Finally, the findings of qRT-PCR supported the reliability of the proposed risk model.

**Conclusion:**

The study identified and established the 8-CRL model, which represents a novel pathway of lncRNA regulation of cuproptosis in OSCC. This model provides guidance for the prognosis and treatment of OSCC and offers a new insight into immune escape and tumor drug resistance.

**Supplementary Information:**

The online version contains supplementary material available at 10.1186/s41065-024-00311-5.

## Background

 As a malignant tumor, oral squamous cell carcinoma (OSCC) specifically occurs in the oral cavity and is among the most malignant tumors of the head and neck, with more than 50% of head and neck squamous cell carcinoma (HNSCC) being OSCC [1]. In 2020, worldwide, OSCC will account for 177,757 deaths (1.8% of all cancers) and 377,713 new cases (2% of all cancers) [2]. Although great progress has been made in immunotherapy and targeted therapy recently, it is often difficult to achieve satisfactory results with anti-tumor drugs in the treatment of OSCC due to the acquisition of tumor resistance [3]. One of the major challenges in the treatment of OSCC today is drug resistance due to the escape of OSCC cells from the regulated cell death (RCD) pathway, and the discovery of a novel RCD process -cuproptosis is expected to overcome this resistance mechanism [4]. Therefore, it is vital to determine the molecular mechanisms related to cuproptosis and OSCC occurrence and progression, explore new ideas for OSCC drug resistance, and detect novel prognostic risk models that can be effectively and reliably applied to manage this type of cancer.

As an essential trace metal, copper makes an important impact on human life activities [5]. Regulation of RCD is known to be critical in identifying cell fate, whereas the mechanism of cytotoxicity and cell death triggered by excessive copper exposure has not been fully elucidated. In recent years, Tsvetkov et al. demonstrated for the first time in the study of Science that there is a copper-dependent and regulated cell death in human cells, a novel RCD mode that relies on mitochondrial respiration but shows difference from known mechanisms of regulated cell death (containing apoptosis, necroptosis, pyroptosis, ferroptosis, etc.)and named this novel copper-dependent cell death mode as “cuproptosis“ [6]. In this process, copper ions directly bind to the fatty acyl components in the tricarboxylic acid cycle pathway. This contributes to abnormal aggregation of fatty acyl proteins and loss of iron-sulfur cluster proteins, which induces proteotoxic stress and finally results in cell death. Copper accumulation is engaged in vital characteristics of cancer progression, containing proliferation, metastasis, and angiogenesis [7]. It has been indicated that higher levels of copper are related to malignancies compared to normal tissues, such as breast cancer [8] and oral cancer [9]. It has been previously confirmed that serum copper levels are notably higher in oral cancer patients than in healthy controls and that excess copper in serum shows an association with oral cancer risk [9]. The copper-enriched nature of tumor tissues is emerging as an attractive target for developing anticancer drugs. Moreover, cuproptosis offers novel ideas for treating multiple tumors, especially in the area of tumor drug resistance. We need to study the biomarkers of cuproptosis in OSCC further to offer a novel direction for treating OSCC.

With the continuous development and advancement of bioinformatics and genomics, a class of long non-coding RNAs (LncRNAs) with the length of over 200 nucleotides is aberrantly expressed in OSCC [10]. LncRNAs affect the biological processes of OSCC cells, like proliferation, migration, and invasion, by participating in epigenetic regulation and post-transcriptional modifications. Existing research has indicated that lncRNAs are engaged in diverse biological processes in cancer, including epigenetic regulation, metabolic disorders, chemoresistance, and immune escape [11]. More and more scholars have reported that lncRNAs act as prognostic biomarkers or potential targets for targeted therapies and are involved in the progression of OSCC [12–14]. Many studies from the direction of lncRNA regulation as a therapeutic and prognostic point for OSCC in clinical first-line radiotherapy regimens to improve tumor resistance to drugs to enhance treatment outcomes. Additionally, lncRNAs make a vital role in copper metabolism as epigenetic regulators, and thus they can be used to help study tumor progression. Given the importance of lncRNAs in cuproptosis, new methods to predict the prognosis of OSCC patients become possible. Nevertheless, cuproptosis-related lncRNAs (CRLs) in OSCC have not been systematically investigated. Therefore, exploring potential targets of cuproptosis mechanisms in OSCC and explaining key CRLs with prognostic significance in OSCC patients deserve future investigation which is important for the study of OSCC mechanisms as well as prognosis and clinical treatment. Herein, through bioinformatics analysis and experimental validation, the present study attempted to build a cuproptosis-related lncRNA prediction model in order to determine the prognosis, immune response, as well as targeted drug sensitivity in OSCC.

## Results

### Identification of Cuproptosis-Related LncRNAs (CRLs) in OSCC

**Fig. 1:**
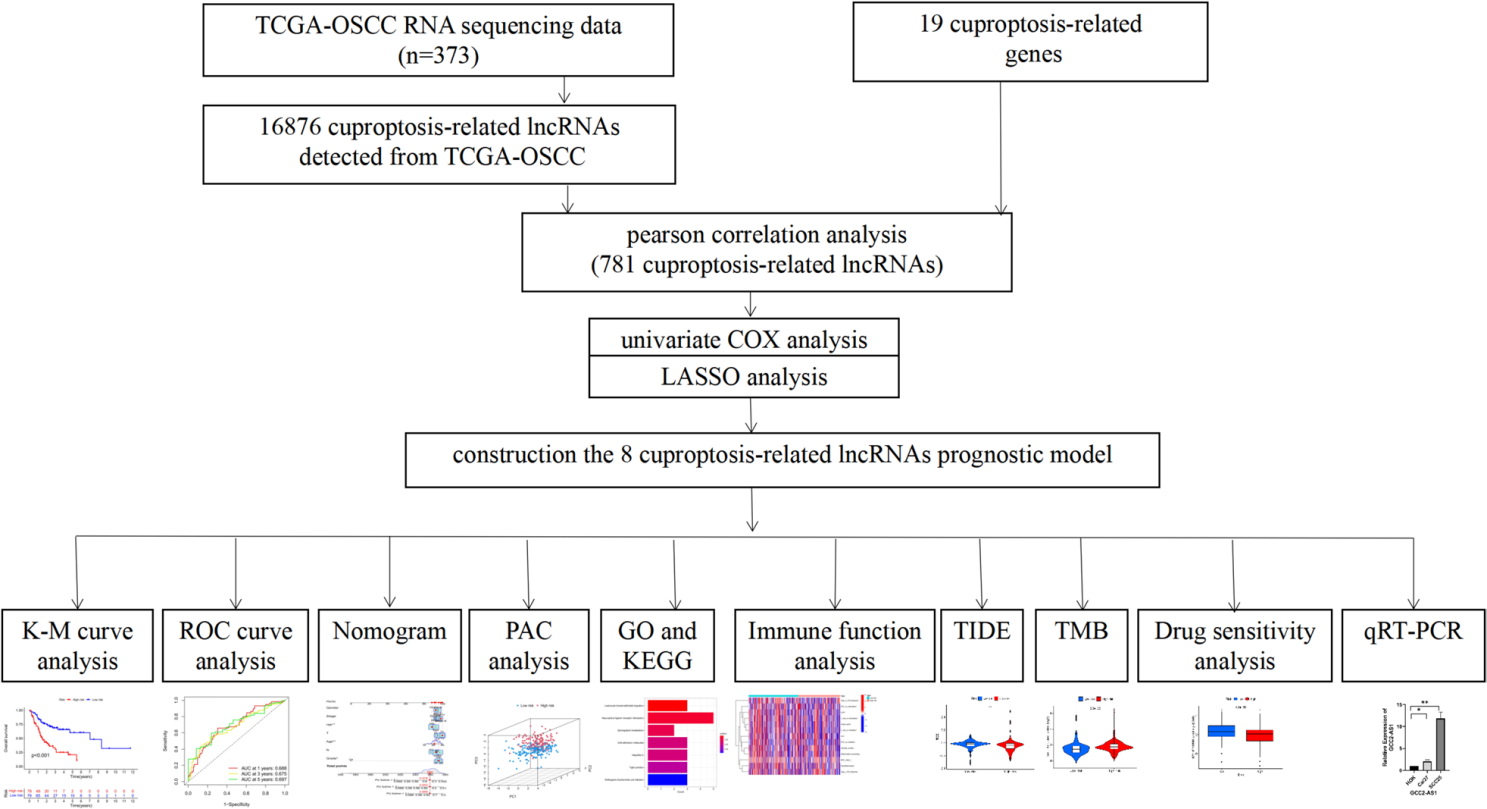
Workflow of cuproptosis-related lncRNA prognostic model establishment and verification

**Fig. 2:**
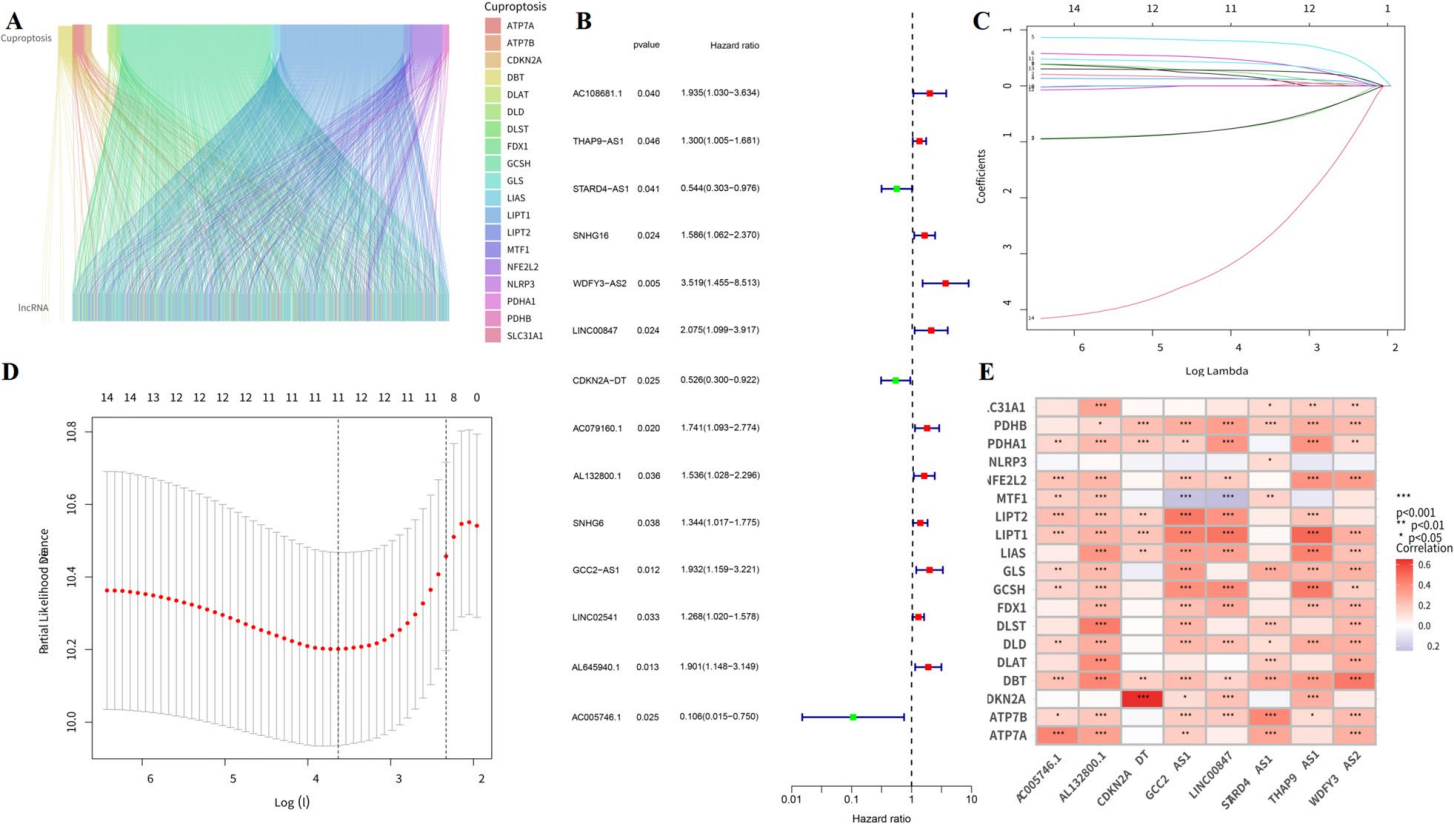
The detection of cuproptosis-related lncRNAs in individuals with OSCC. **A** Sankey diagram representing the relation between 19 cuproptosis-related genes and lncRNAs related to cuproptosis. **B** To screen CRLs in relationship to clinical prognosis, univariate Cox regression analysis was employed. **C, D** Following the LASSO analysis to select the optimal value of the penalty λ. **E** Heatmap shows co-expression analysis of 8 prognostic CRLs and 19 CRGs. Besides, the level of the association is shown in the color of each unit. *P < 0.05, **P < 0.01, ***P < 0.001. *CRG* Cuproptosis-related gene, *LASSO* Least absolute shrinkage and selection operator, *CRL* Cuproptosis-related lncRNA

Figure 1 displays the workflow. This study retrieved transcriptome RNA-Seq data from 373 TCGA-OSCC cases, including 341 OSCC tissues and 32 nearby normal tissues, with pertinent clinical information. The present study included samples with adequate clinical data for further analysis. There were 16,876 lncRNA detected from the TCGA-OSCC gene expression file based on the GTF annotation file for human signatures. The expression data of 19 cuprotosis-relevant genes (CRGs) in OSCC samples were also retrieved from TCGA database. Pearson’s correlation analysis yielded 781 lncRNAs cuproptosis-related lncRNAs with significant correlation (*R* > 0.4, *p* < 0.001). The Sankey diagram reveals the degree of connection between the 19 cuproptosis-related mRNAs and 781 lncRNAs (Fig. 2A). With the aim of examining the prognostic ability of these CRLs, this study classified the TCGA-OSCC data (*n* = 316) into a training group (*n* = 158) and a test group (*n* = 158) randomly. Clearly, the clinical features containing age, gender, and TNM stage tended to present no statistically significant difference (Table 1, *P* > 0.05), indicating no bias in sample grouping. To identify lncRNAs with prognostic significance, single-factor Cox analysis was adopted for detecting 14 CRls (|cor|>0.4 and *P* < 0.001) (Fig. 2B). Subsequently, LASSO regression analysis detected 11 CRLs (Fig. 2C.D). Multifactor Cox analysis further determined 8 CRLs with prognostic significance. Figure 2E indicates the co-expression correlation between these 8 CRLs and the 19 CRGs.

**Table 1:** The clinical features of individuals with OSCC on the basis of the TCGA database

Covariates	Type	Entire	Testing	Training	*P* value
Age	< = 65	200(63.29%)	105(66.46%)	95(60.13%)	0.2935
	> 65	116(36.71%)	53(33.54%)	63(39.87%)	
Gender	FEMALE	95(30.06%)	48(30.38%)	47(29.75%)	1
	MALE	221(69.94%)	110(69.62%)	111(70.25%)	
Grade	G1	49(15.51%)	27(17.09%)	22(13.92%)	0.8556
	G2	195(61.71%)	94(59.49%)	101(63.92%)	
	G3	63(19.94%)	32(20.25%)	31(19.62%)	
	G4	2(0.63%)	1(0.63%)	1(0.63%)	
	unknow	7(2.22%)	4(2.53%)	3(1.9%)	
Stage	Stage I	20(6.33%)	10(6.33%)	10(6.33%)	0.6784
	Stage II	53(16.77%)	31(19.62%)	22(13.92%)	
	Stage III	55(17.41%)	27(17.09%)	28(17.72%)	
	Stage IV	153(48.42%)	75(47.47%)	78(49.37%)	
	unknow	35(11.08%)	15(9.49%)	20(12.66%)	
T	T0	1(0.32%)	1(0.63%)	0(0%)	0.435
	T1	32(10.13%)	19(12.03%)	13(8.23%)	
	T2	99(31.33%)	52(32.91%)	47(29.75%)	
	T3	57(18.04%)	24(15.19%)	33(20.89%)	
	T4	100(31.65%)	52(32.91%)	48(30.38%)	
	unknow	27(8.54%)	10(6.33%)	17(10.76%)	
M	M0	114(36.08%)	57(36.08%)	57(36.08%)	1
	unknow	202(63.92%)	101(63.92%)	101(63.92%)	
N	N0	116(36.71%)	68(43.04%)	48(30.38%)	0.2366
	N1	46(14.56%)	23(14.56%)	23(14.56%)	
	N2	97(30.7%)	44(27.85%)	53(33.54%)	
	N3	3(0.95%)	1(0.63%)	2(1.27%)	
	unknow	54(17.09%)	22(13.92%)	32(20.25%)	

### Construction of risk model for CRLs

**Fig. 3:**
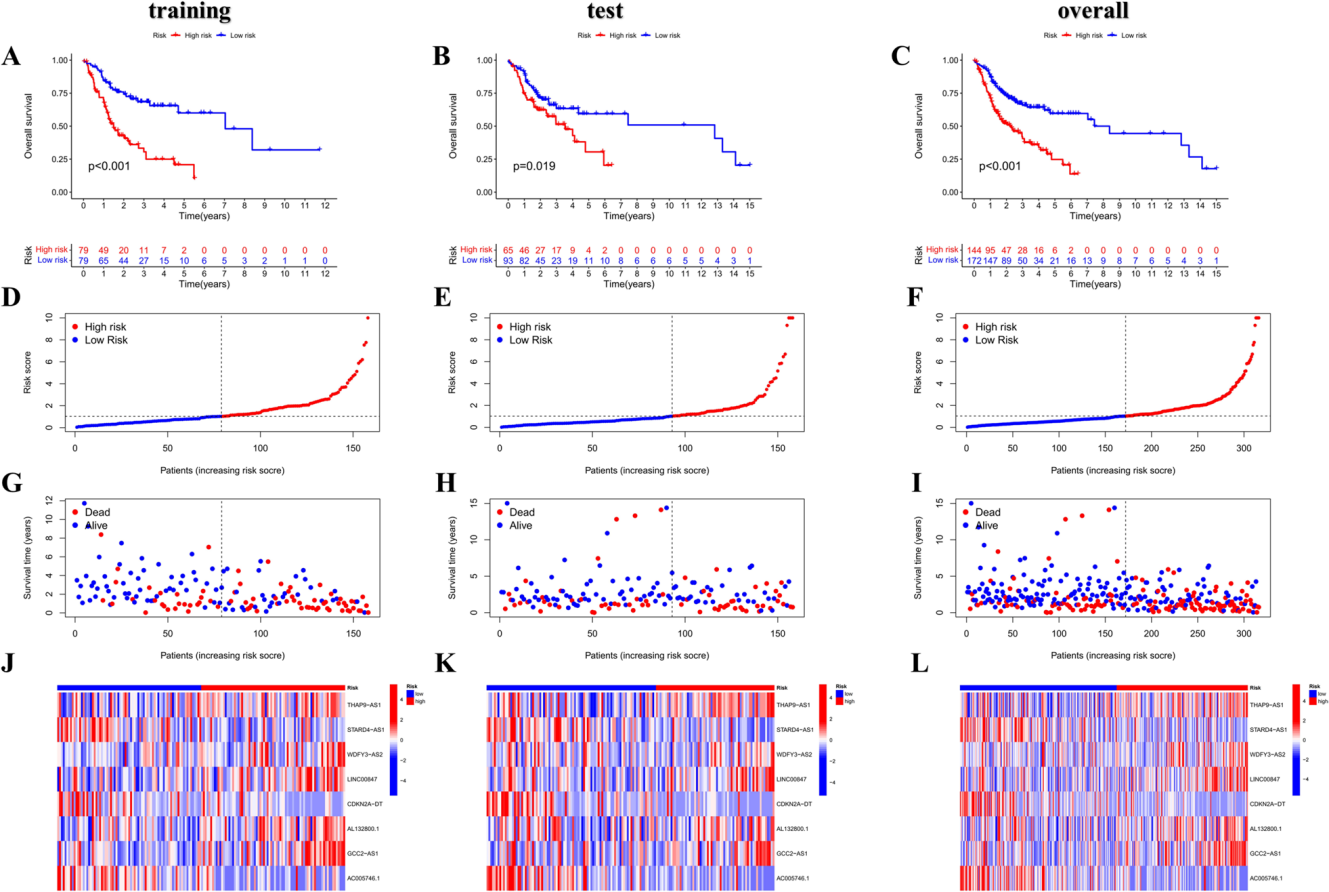
Establishment of risk models for 8 lncRNAs related to cuproptosis in OSCC. **A**-**C** Survival analysis of low and high-risk groups in the training, test, and overall group. **D**-**F** Risk score curves. **G**-**I** Scatter plots of survival status. **J**-**L** Heatmap of the levels of 8 lncRNAs

**Fig. 4:**
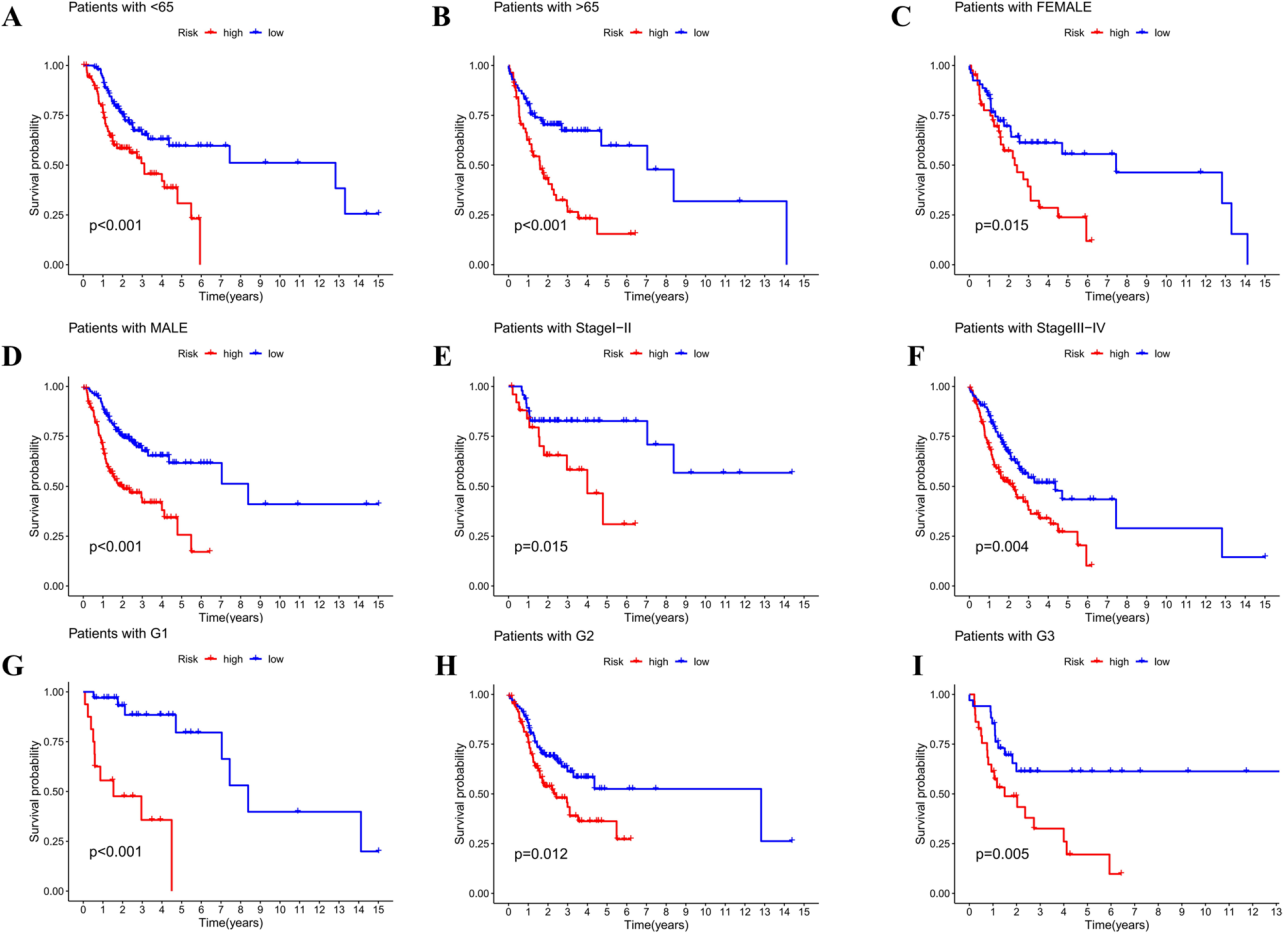
Survival curves for model validation. The prognostic model can be adopted for different clinical subgroups of OSCC: **A**,** B** Age (≤/>65 years). **C**,** D** Gender (female/male). **E**,** F** Clinical stage (I-II/III-IV). **G**,** H**,** I** Pathological grade (1, 2, 3), *P* < 0.05

To assess the prognostic risk of OSCC patients, a risk model was established with eight cuproptosis-related lncRNAs. Each OSCC patient in the TCGA database was set a risk score based on the formula: Risk score = THAP9-AS1 × 0.285166029638806 + STARD4-AS1 × (-1.0021836589916) + WDFY3-AS2 × 1.07158530871364 + LINC00847 × 0.723466955195127 + CDKN2A-DT × (-1.01755473896558) + AL132800.1 × 0.382836439019932 + GCC2-AS1 × 0.492465300792503 + AC005746.1 × (-3.68465588796498). Through the median risk value, we categorized OSCC samples into high-risk and low-risk groups. Next, the present study assessed the prognostic value of the 8-CRL model. All three analyses in the training, test, and overall group (Fig. 3A-L) obtained the same trend findings. The K-M curve revealed that the survival rate of the low-risk group was notably higher than that of the high-risk group (training group: *P* < 0.001; test group: *P* = 0.019; overall group: *P* < 0.001). According to the risk score curve and scatter plot of survival status (blue dots indicate survival and red dots suggest death), with the increased risk, the sample mortality rate significantly elevated, and most deaths were concentrated in the high-risk population. The risk heat map showed five risk lncRNAs (THAP9-AS1, WDFY3-AS2, LINC00847, AL132800.1, and GCC2-AS1) presented notable up-regulation in the high-risk subgroup, while three protective lncRNAs (STARD4-AS1, CDKN2A-DT, and AC005746.1) presented significant down-regulation (Figure 10 in Appendix). All these suggested that the 8-CRL model has a good predictive ability. By contrast, the mortality rate of the high-risk group was shown to be higher. We investigated the correlation between the predictive parameters of OSCC patients and OS, as well as classified according to various clinical and pathological characteristics containing age, gender, grading, and staging. By contrast, patients in the high-risk group revealed notably shorter OS in all key clinical features (Fig. 4). This indicates that our model is suitable for early and late-stage OSCC patients and patients of different genders and staging. The 8-CRL model serves as a valuable prognostic model for OSCC.

### Validation of the accuracy of the prognostic risk model for CRLs

**Fig. 5:**
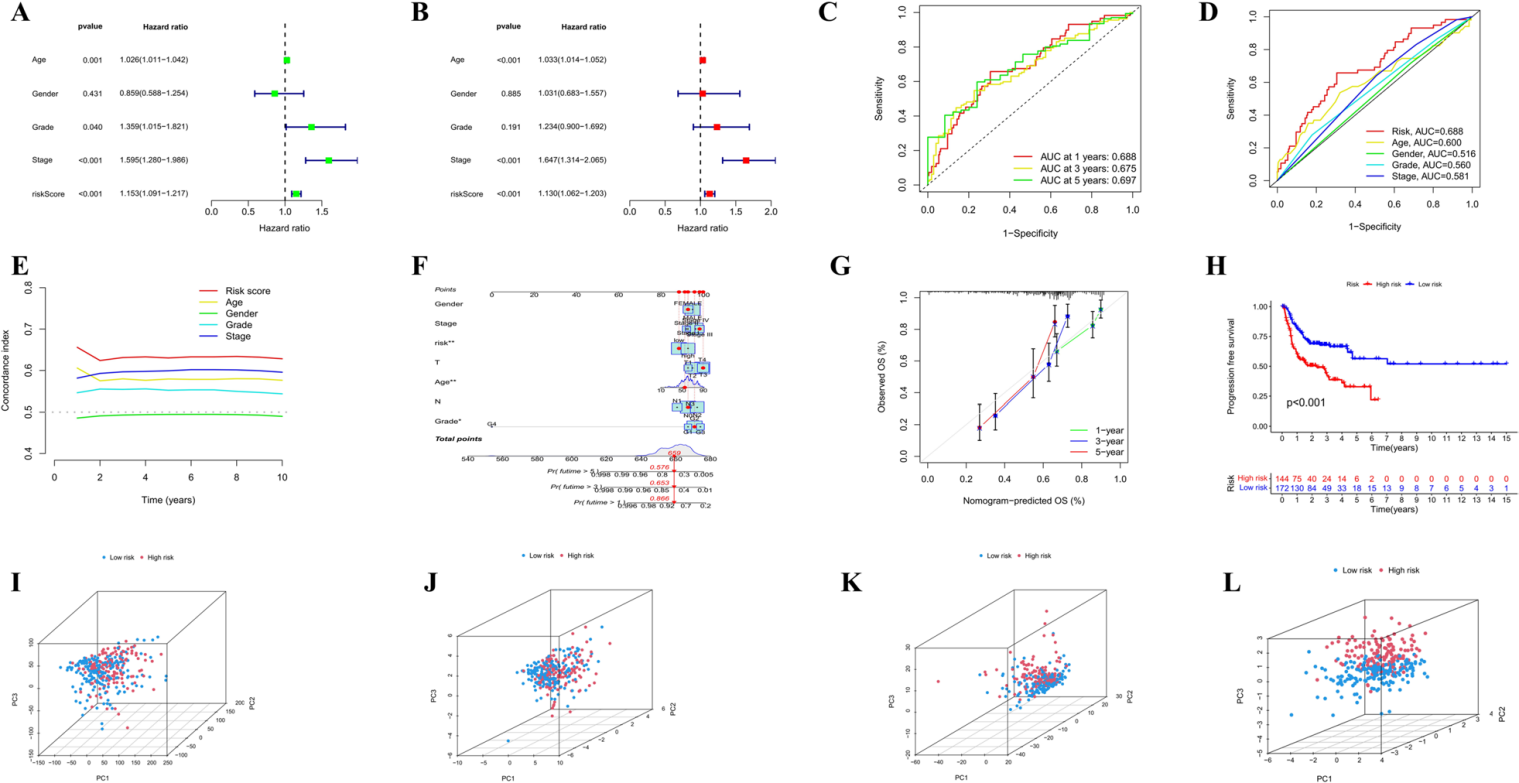
The validation of the accuracy of the Prognostic Risk Model. **A, B** Univariate/multivariate Cox analysis of risk score and clinical factors. **C** Time-dependent ROC curve AUC for survival prediction using risk score. **D** Comparison of AUC of ROC curves for prognostic accuracy between risk score and the clinical characteristics. **E** C-index curve comparison of prognostic accuracy between risk score and clinical factors. **F** The establishment of nomogram for determining the survival of OSCC individuals. **G** Calibration curves corresponding to column line plots were adopted to determine the concordance between 1-, 3-, and 5-year OS. **H** Kaplan–Meier curves displaying the progression-free survival. PCA between low- and high-risk populations (**I**) All gene set. **J** Cuproptosis gene set. **K** CR LncRNAs set. **L** 8-CRL prognostic model set. *ROC* Subject working characteristic curve, *PCA* Principal component analysis, *AUC* Area under the curve

To explore whether the 8-CRL model is an independent prognostic predictor for OSCC patients, Cox regression analysis was employed. Using Univariate Cox regression analysis, age, stage, and risk score showed relationship to OSCC prognosis (*P* < 0.001) (Fig. 5A). Based on multivariate Cox regression analysis, age, stage, and risk score were independent prognostic indicators for OSCC (*P* < 0.001) (Fig. 5B), and the findings suggest that the constructed model can be applied as an independent prognostic factor to differentiate from the effects of other clinical characteristics. For the purpose of evaluating the accuracy and specificity of the risk score, we adopted the area under the ROC curve(AUC). Besides, the 1, 3, and 5-year AUCs for all samples were separately 0.688, 0.675, and 0.697 (Fig. 5C). Compared with the AUCs of age, sex, tumor grade, and clinical stage in the ROC curve, the risk score AUC remained the highest (Fig. 5D), indicating higher predictive efficiency. As time passed, the C-index of the risk score was always higher than that of other clinical components (Fig. 5E). It is demonstrated that the prognosis of cuproptosis-related lncRNA has a better predictive ability than other clinical and pathological features. A Nomogram was developed with age, sex, grade, stage, and risk score factors, aiming to predict the survival rates of OSCC patients at 1, 3, and 5 years. When the patient is female, 50 years old, in the low-risk group, stage IV, with T4, N3, and grade G2, the total score calculated according to the corresponding points in the line graph is 659, corresponding to a 5-year survival rate of 0.576, a 3-year survival rate of 0.653, and a 1-year survival rate of 0.866 (Fig. 5F). In addition, the calibration plots for 1, 3, and 5 years are close to the gray solid line, suggesting good predictive ability (Fig. 5G). The K-M survival curve demonstrated that the progression-free survival(PFS)of OSCC patients in the low-risk group was notably better (Fig. 5H), suggesting that the model is capable of effectively distinguishing high-and low-risk groups. Then, PCA analysis was performed with the entire gene sequencing data of the TCGA-OSCC cohort, 19 cuproptosis-related genes, 781 cuproptosis-related lncRNAs and 8-CRL risk prognostic model (Fig. 5I-L). In addition, the significant and stable differences in distributing the two groups on the basis of the risk model fully suggested that the risk model is capable of efficiently finding high-risk patients, proving the model’s accuracy.

### Functional enrichment analysis based on risk model

**Fig. 6:**
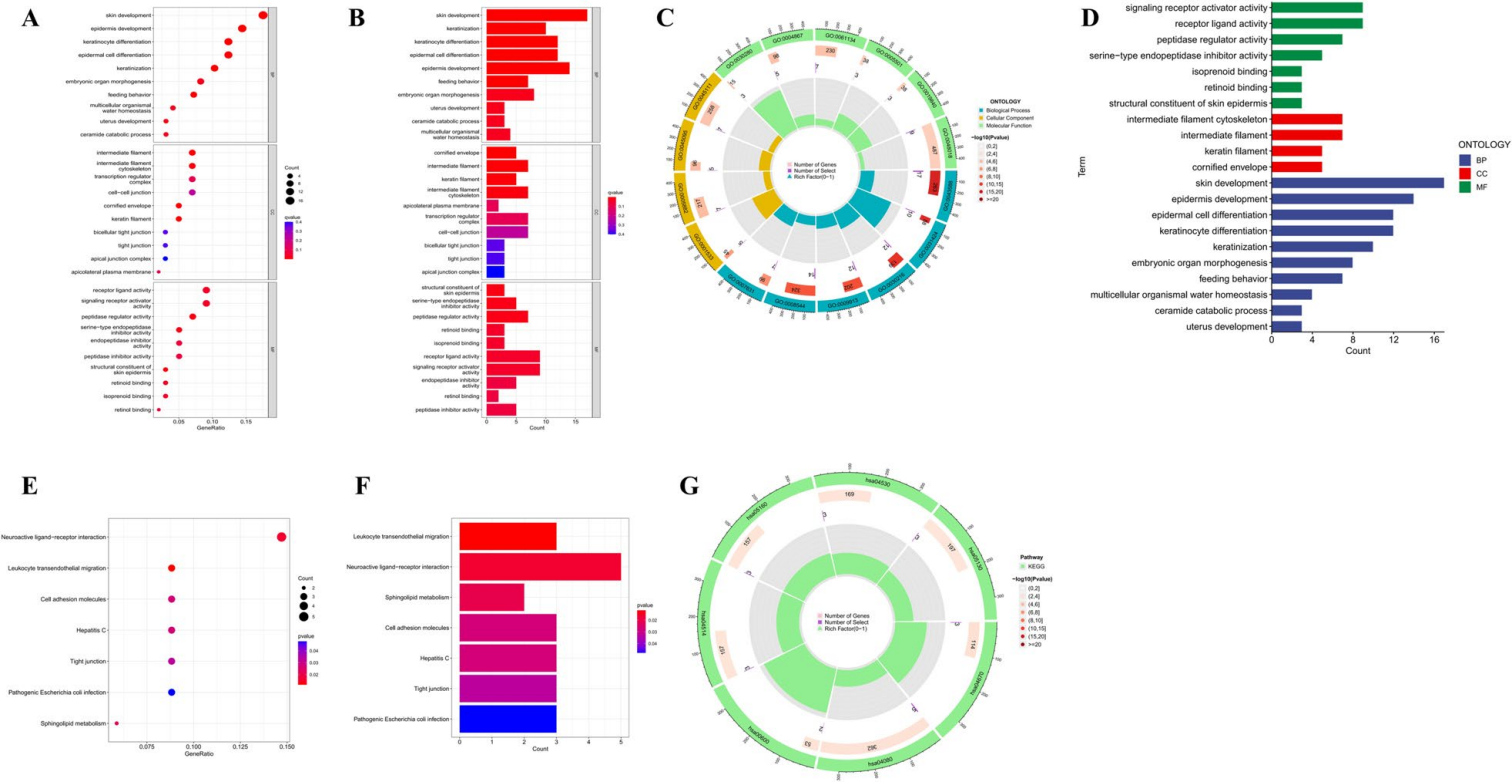
GO and KEGG analysis. **A** Bubble plot. **B, D** Bar plot. **C** Circular plot, biological process (BP), cellular component (CC), and molecular function (MF) of DEGs obtained from GO analysis between the two groups. **E** Bubble plot. **F** Bar plot. **G** Circular plot, DEGs were obtained from KEGG analysis between the two groups. DEG: differentially expressed gene; GO: gene ontology; KEGG: Kyoto Encyclopedia of Genes and Genomes

In this study, a total of 139 differentially expressed genes (DEGs) were identified between high-risk and low-risk groups by differential expression analysis (Supplement Table S1). Next, KEGG and GO enrichment analysis of these DEGs were performed to explore the potential biological function differences in the prognosis difference of OSCC patients in different risk groups. GO analysis suggested that DEGs were notably enriched in biological processes (BP), covering skin development, epidermal development, epidermal cell differentiation, keratinocyte differentiation, and keratinizing ability. Concerning cellular components (CC), the DEGs were notably enriched in the intermediate filament cytoskeleton, intermediate filament fibers, keratin filaments, and keratinizing envelope. Concerning molecular function (MF), these DEGs were significantly enriched in signaling receptor activator activity, receptor ligand activity, peptidase regulatory activity, and serine-type endopeptidase inhibitor (Fig. 6A, B, D). KEGG results suggest that these DEGs may be associated with neuroactive ligand-receptor interactions, leukocyte transendothelial migration, and sphingolipid metabolism (Fig. 6E, F), suggesting that activation of these pathways may increase the risk of death in patients. The circular graphs indicate the number of genes, the number of enrichments, and the gene ratios in GO and KEGG enrichment analysis (Fig. 6C, G).

### Comparison of immune function and TIDE score between high and low-risk groups

**Fig. 7:**
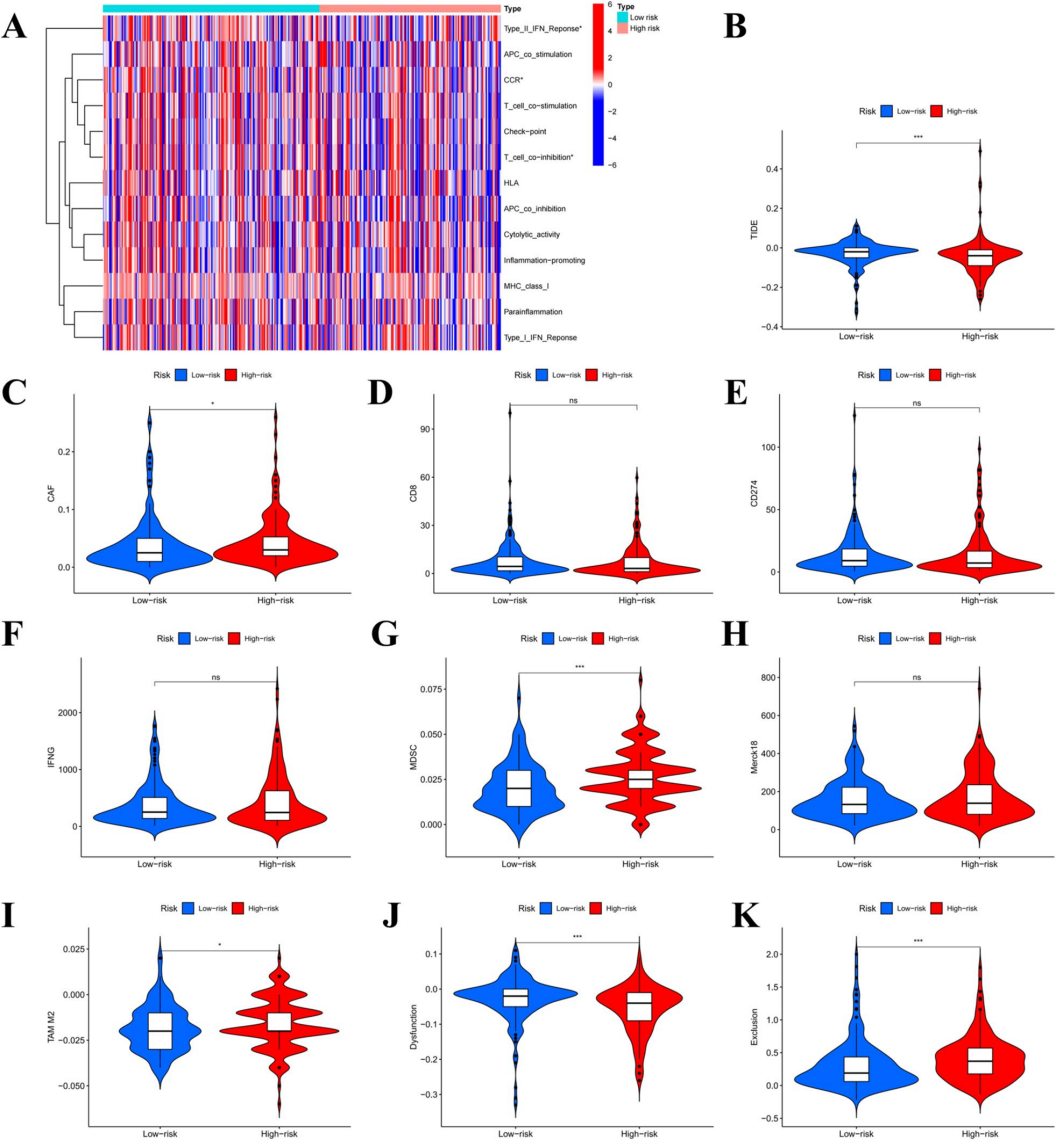
Immune function analysis for evaluating immune status in both risk groups. **A** Heat map representing immune function distribution. **B** TIDE score. **C** CAF score. **D** CD8 score. **E** CD274 score. **F** IFNG score. **G** MDSC score. **H** Merck18. **I** TAM M2 score. **J** Dysfunction. **K** Exclusion. *P < 0.05, **P < 0.01 and ***P < 0.001. *CAF* Cancer-associated fibroblast, *CD* Cluster of differentiation, *IFNG* Interferon-gamma, *MDSC* Myeloid-derived suppressor cell, *TAM* Tumor-associated macrophages, *TIDE* Tumor immune dysfunction and exclusion

With the aim of exploring the immune status of the low and high-risk groups, immune-related functions were analyzed. Clearly, the findings revealed differences in type-II-IFN-response and chemokine receptor expression (CCR) (Fig. 7A). We obtained TIDE scores and scores for CAF, IFNG, CD8, CD274 (PD-L1), MDSC, T-cell dysfunction, Merck18, TAM M2, and T-cell exclusion from the TIDE website. The TIDE score was higher in the high-risk group, implying more obvious tumor immune escape and poor response to immune therapy in the high-risk group (Fig. 7B). The present work explored the distribution of these scores between the risk groups in more detail and found that the CAF, T-cell Dysfunction, and MDSC scores were higher in the high-risk group, whereas the TAM M2 and T-cell exclusion scores were shown to be higher in the low-risk group (Fig. 7C-K).

### Tumor Mutation Burden (TMB) analysis through risk model comparison

**Fig. 8:**
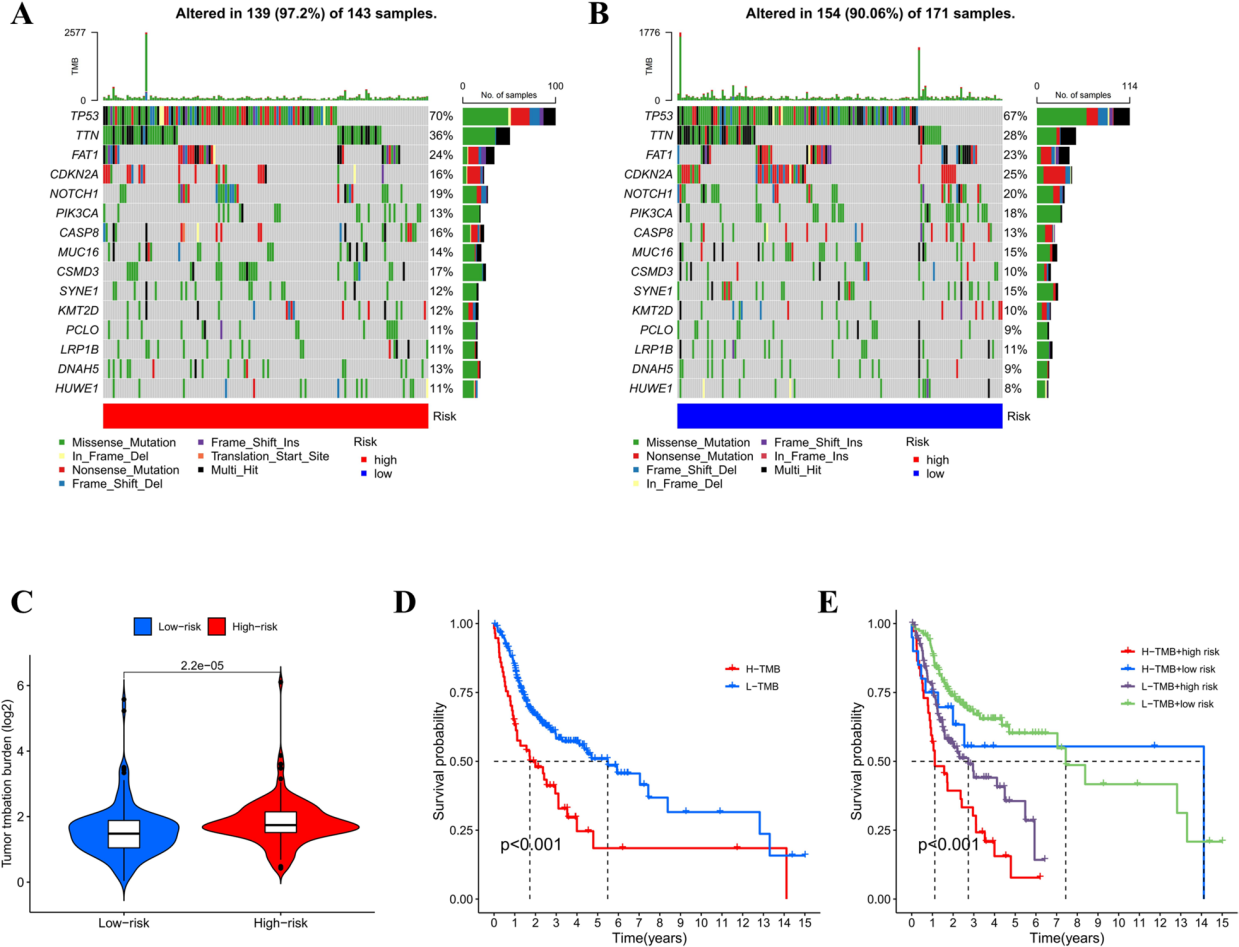
TMB analysis through risk model comparison. Waterfall plots of death-related gene mutations in a high-risk group (**A**) and low-risk group (**B**) of OSCC patients. **C** Violin plot of the difference in TMB. **D** Kaplan-Meier curves of OS for high TMB and low TMB subgroups. **E** Kaplan-Meier curves of OS for TMB + risk. *KM* Kaplan-Meier, *OS* Overall survival, *TMB* Tumor mutational burden

Firstly, the difference in tumor mutation burden (TMB) between the two groups of patients was evaluated. The top 15 genes which had the highest mutation frequencies are displayed in Fig. 8A and B. The waterfall plot reveals that the top 3 mutated genes in OSCC samples are TP53, TTN, and FAT1. Comparatively, the mutation frequency of most genes in the high-risk group is higher. To study the prognostic value of TMB, OSCC samples were classified into high and low TMB subgroups in line with the median TMB score. In addition, survival analysis was also conducted in this study. The median survival time (MST) of the high TMB subgroup was notably higher in relative to that of the low TMB subgroup (*P* = 2.22 e-0.6, Fig. 8C). Thus, the risk model based on 8-CRl relates to TMB. Joint survival analysis of TMB and risk score revealed that the MST difference among the four groups showed statistical significance(*P* < 0.001, Fig. 8D.E). We noticed that the low TMB + low-risk group exhibited the best prognosis. In contrast, the high TMB + high-risk group exhibited the worst prognosis, highlighting the significant synergistic effect between these two indicators.

### Screening of potential drugs for OSCC based on the 8-CRL model

**Fig. 9:**
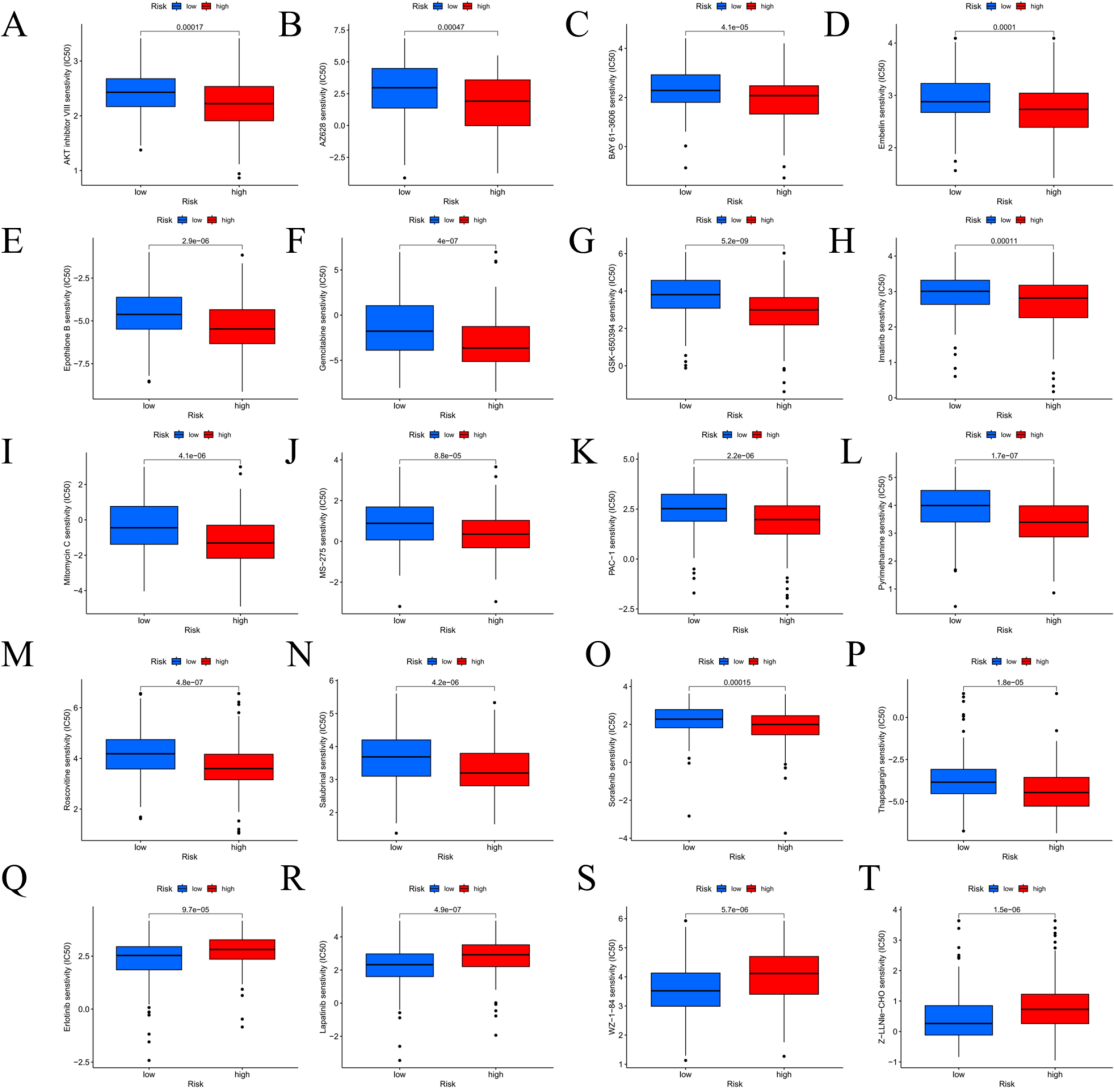
Drug sensitivity analysis using the 8-CRL prognostic model in OSCC

**Fig. 10:**
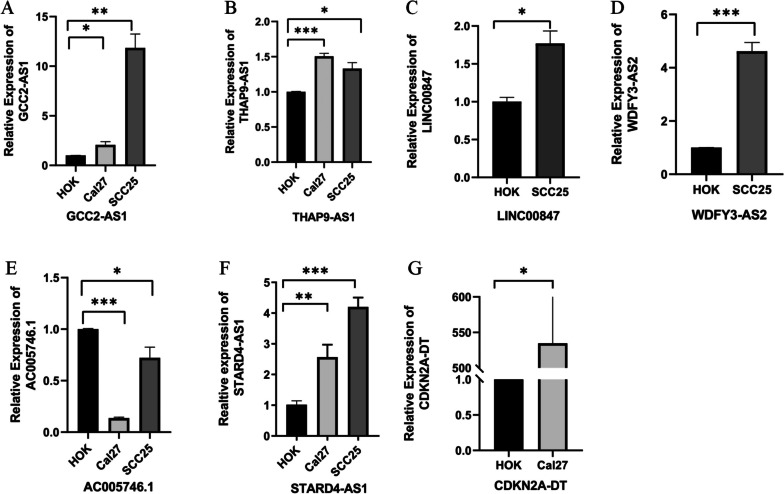
Level of 8-CRL in normal oral keratinocytes and OSCC cell lines identified through RT-qPCR. *P < 0.05, **P < 0.01, and ***P < 0.001. *qRT-PCR* Quantitative reverse transcription–polymerase chain reaction

OSCC has relatively poor sensitivity to various anti-tumor drugs, which limits its widespread application. Given the notable differences in prognosis between the two groups of OSCC patients, we determined to further screen potential drugs, aiming to obtain targeted therapy better. By contrasting the IC50 values of some commonly seen drugs in different risk populations, it could be found that, to some extent, the risk score of OSCC patients can affect their sensitivity to drugs. The IC50 values of 16 potential drugs, containing Gemcitabine and Sorafenib, were notably lower in the high-risk group (Fig. 9A-P), implying that they may be candidates for high-risk group OSCC patients. Comparatively, the IC50 values of four potential drugs, erlotinib and lapatinib, were significantly down-regulated in the low-risk group (Fig. 9Q-T), suggesting that low-risk OSCC patients may be more sensitive to these four drugs. Detailed descriptions of the drugs are shown in Table 2.

**Table 2:** List of 16 antitumor drugs that are more sensitive in high-risk patients and 4 antitumor agents with more sensitivity in patients with low risk

16 antitumor drugs that are more sensitive to patients in the high-risk group	
Antitumor drugs	Description
AKT inhibitor	AKT inhibitor
AZ628	Raf inhibitor
BAY 61–3606	Syk inhibitor
Embelin	IAP inhibitor
Epothilone.B	Macrolide antitumor factors
Gemcitabine	Gemcitabine
GSK-650394	SGK inhibitor
Imatinib	tyrosine kinase inhibitor
Mitomycin C	Mitomycin C
MS-275	Entinostat HDAC inhibitor
PAC-1	Caspase activator
Pyrimethamine	Ethambutol
Roscovitine	CDK inhibitor
Salubrinal	eIF2α dephosphorylation inhibitor
Sorafenib	The serine/threonine kinase activities of RAF-1 and B-Raf and tyrosine kinase activities of VGFR-2, VEGF-3, PDGF-β, KIT and FLT-3 receptors were inhibited
Thapsigargin	Tunicamycin,endoplasmic reticulum stress inducer
4 antitumor drugs that are more sensitive to patients in the low-risk group	
Antitumor drugs	Description
Erlotinib	HER1/EGFR tyrosine kinase inhibitor
Lapatinib	Lapatinib
WZ-1–84	PARP inhibitor
Z-LLNle-CHO	γ-Secretase inhibitor I

### Validation of 8-CRL expression levels in OSCC

To further explore the expression of CRL, RT-qPCR analysis demonstrated that compared with human normal oral keratinocyte cells (HOK), the expression levels of GCC2-AS1, THAP9-AS1, LINC00847, and WDFY3-AS2 were upregulated, and AC005746.1 presented downregulation in OSCC cell lines (Cal27 and SCC25). These lncRNAs levels conformed to the findings of bioinformatics analysis. Nevertheless, STARD4-AS1 and CDKN2A-DT were highly expressed in OSCC cell lines. RT-qPCR detection of CRL expression provided some experimental evidence, although it did not completely conform to the results of bioinformatics analysis. Overall, 8-CRL may make a vital impact on the occurrence and progression of OSCC.

## Discussion

The pathogenesis of OSCC closely relates to immunosuppression and escape, with cancer cells evading immune surveillance and antitumor immune responses through immunosuppressive cytokine accumulation, impaired cellular activity and T-cell depletion [15]. The importance of copper in immune infiltration has also been reflected in some recent correlation studies. TAN found that the copper chelating agent on macrophages could eliminate the presentation of PD-L1 mediated by lysyl oxidase-like 4(loxl4), thus inhibiting the immune escape of cells [16]. Inducing cuproptosis in cancer cells activates the ability of immune cells and can be used as a strategy to enhance immunotherapeutic activity. Numerous studies have confirmed that lncRNAs, as epigenetic factors, can regulate cuproptosis, and lncRNAs make a vital impact on the occurrence, development, and immune escape of OSCC [17]. With the emergence of some new lncRNA targeting technologies combined with immune checkpoint therapy, they offer a novel strategy for treating OSCC. However, up to now, only a few studies have studied cuproptosis-related lncRNAs in HNSCC. Given the particularity of OSCC, it is essential to study the association between survival prognosis and risk of cuproptosis-related lncRNAs in OSCC.

With univariate Cox analysis, LASSO regression, and multivariate Cox analysis, totally 8 lncRNAs related to cuproptosis (THAP9-AS1, STARD4-AS1, WDFY3-AS2, LINC00847, CDKN2A-DT, Al132800.1, GCC2-AS1, and AC005746.1) were found to build the risk prognosis model. In the subsequent research analysis, the superior predictive power of the risk model is verified. In the 8-CRLs studied, THAP9-AS1, CDKN2A-DT, GCC2-AS1, AL132800.1, STARD4 − AS1, and WDFY3-AS2 were reported to be biomarkers related with cuproptosis in HNSCC [18–23]. LINC00847 is engaged in the regulation of pyroptosis in the occurrence and development of OSCC [24]. In addition, WDFY3-AS2 has been found to make an important impact on diverse cancers by regulating ferroptosis [25]. Silencing WDFY3-AS2 notably hinders the proliferation, migration and invasion of oral squamous cells but accelerates cell apoptosis and can be applied to be a new therapeutic target and prognostic factor for oral squamous cell carcinoma [26]. Apparently, these lncRNAs are involved in diverse regulatory cell death pathways, containing cuproptosis, pyroptosis, ferroptosis, and apoptosis, suggesting that different kinds of regulated cell death can be viewed to be a single, coordinated cell death system where the pathways are greatly interconnected and can make flexible compensation for each other. 8-CRL markers provide new targets for future mechanistic studies.

With the use of the median risk score, this study classified OSCC patients into a high-risk group and a low-risk group to demonstrate the predictive power of the model. The findings of risk analysis, survival analysis, and 1, 3, and 5-year time-dependent ROC analysis between the two risk groups well showed the validity of the 8-CRL model prediction. Univariate and multivariate Cox regression analyses verified that risk score was an independent prognostic factor for OSCC patients in the training, test, and overall group. In addition, we further combined the model with independent prognostic factors (sex, age, grading, and staging) to form a nomogram with better predictive power for overall survival, suggesting that the prediction using the 8-CRL model was more accurate. We adopted GO and KEGG enrichment analysis for investigating the mechanisms related to the risk model. It was shown that DEGs were mainly enriched in pathways related to leukocyte transendothelial migration and sphingolipid metabolism, suggesting that 8-CRL may regulate OSCC progression by affecting immune infiltration levels and metabolism. It is suggested that copper imbalance can affect mitochondrial respiration and lead to metabolic changes inducing RCD and copper-dependent cell death [27], providing clues for the toxic damage related to copper metabolic dysfunction and the potential use of anti-OSCC.

Immune checkpoint inhibitors (ICIs) have opened a revolutionary era in cancer immunotherapy and play a crucial role in immune escape surveillance [28, 29]. It is well known that the TIDE algorithm is adopted for evaluating the clinical response to ICI therapy. A higher TIDE score indicates a greater likelihood of immune escape, implying a restricted response and shorter survival time for patients subjected to treatment with ICI. In the present study, TIDE scores were higher in the high-risk group, whereas no obvious differences were observed in CD274 (PD-L1) scores. However, higher TIDE scores were found to be linked to lower responsiveness to anti-PD-1 therapy [30]. PD-L1 expression levels, degree of immune cell infiltration, and tumor mutational burden (TMB) are predictors of OSCC immunotherapy efficacy. TMB, which is the total number of somatic mutations per megabase (Mb) in the tumor genome, relates to the emergence of neoantigens triggering tumor immunity [31]. Recent studies have indicated that individuals with high TMB are more probably to produce potent biomarkers of anti-PD-1/PD-L1 therapeutic response when being subjected to treatment with ICIs [32]. In cancers such as HNSCC and Bladder cancer, lower TMB had longer OS [33, 34]. Interestingly, combining TMB with risk score in our study revealed the worst prognosis in the high TMB + high risk group and the best prognosis in the low TMB + low risk group. Therefore, risk score, TIDE, and TMB score can be used jointly to achieve more accurate and higher predictive performance in the future. In the gene mutational differential analysis, we identified the tumor suppressor TP53 gene as the most mutated gene, a finding consistent with recent evidence from genome sequencing analysis [35, 36]. Multiple studies have shown that the polymorphism of the TP53 gene can increase the susceptibility to oral cancer and may be one of the risk factors for oral cancer [37, 38]. This indicates that TP53 may become a novel therapeutic target for OSCC patients.

There is evidence that although chemotherapy is extensively applied in the clinical treatment of OSCC and rapidly inhibits its deterioration, the development of tumor drug resistance is often the main factor limiting the therapeutic effect and prognosis of tumor patients and may lead to tumor recurrence [39]. Therefore, improving the understanding of the mechanisms of tumor drug resistance will help the treatment of oral cancer and is the key to improving tumor prognosis. Many current studies have been conducted in the direction of lncRNA regulation as a therapeutic and prognostic point for oral cancer to improve tumor resistance to drugs in clinical first-line radiotherapy regimens to improve treatment outcomes [40]. Copper chelates are currently used as antitumor agents in combination with platinum-based chemotherapeutic agents in many tumor resistance or recurrence, which can enhance the sensitivity of chemotherapeutic agents and produce good synergy [41]. The risk model is used as a possible indicator to predict drug sensitivity and highlights the potential of CRLS in the clinical development of future personalized treatment strategies. We showed that 20 drugs were sensitive; most of them most were targeted therapeutics, and 16 potential drugs in the high-risk group contained gemcitabine and sorafenib. Gemcitabine belongs to the cell cycle-specific antimetabolite class of chemotherapeutic agents [42]. In clinical applications, gemcitabine is used to treat various solid tumors such as pancreatic, head and neck, and breast cancers [43–45]. Pauwels studied the radiosensitizing effect of gemcitabine on tongue cancer CAL-27 cells and found that the mechanism may be related to the promotion of apoptosis [46]. Sorafenib is a multikinase inhibitor that functionally blocks the repair of DNA damage in NF-κB and head and neck squamous cells. The synergistic effect of sorafenib in combination with radiation on human oral squamous cell carcinoma in vitro has also been demonstrated [47, 48], promoting apoptosis and inhibiting migration and invasion of oral cancer cells when used in combination with chemotherapeutic agents [49].

In contrast, there are four potential drugs in the low-risk group, including erlotinib and lapatinib. Erlotinib is a small-molecule tyrosine kinase inhibitor [50]. Studies have shown that erlotinib can inhibit the growth of squamous cell carcinoma of the tongue (SCC-15). In addition, erlotinib inhibited the growth of SCC-15 cells in concert with cisplatin and radiation. EGFR-targeting erlotinib in combination with VEGF inhibitors also showed promising results in phase I and II trials in metastatic and recurrent oral cancers [50]. Lapatinib is a well-tolerated oral EGFR and HER2 inhibitor in cancer patients [51]. Based on the 8-CRL model, we conclude that combining immunotherapy with chemotherapy or other targeted inhibitors will offer a precise and personalized clinical treatment strategy for individuals with OSCC. Although this risk model can provide the choice of potential drugs, clinical application needs to be demonstrated through cell experiments and clinical trials.

To predict the accuracy of our prognostic model, a preliminary RT-qPCR test showed that GCC2-AS1, THAP9-AS1, LINC00847, and WDFY3-AS2 were up-regulated in OSCC cell lines relative to human normal oral keratinocyte lines (HOK). AC005746.1 presented down-regulation in OSCC cell lines, and the levels of these lncRNAs conformed to the findings of bioinformatics analysis. Nevertheless, we noted an abnormally high expression of STARD4-AS1 and CDKN2A-DT in OSCC cells, and Cox regression analysis suggested that it may play a protective role. This seems to contradict the conventional wisdom that genes highly expressed in tumors are oncogenes, and poorly expressed genes are cancer-suppressor genes. In fact, we have missed an important point that tumors result from staged development. For example, TGF-β, which acts at different stages of cancer, may be oncogenic in early stages and pro-oncogenic in late stages [52]. Although the expression of CRL detected by RT-qPCR was not in complete consistence with the findings of bioinformatics analysis, it demonstrated the accuracy of the prediction of our model to a certain extent, and further research is needed. In conclusion, the 8-CRL marker found in our study provides a new target for future mechanism research and a new idea for further in vivo and in vitro experimental research.

Overall, this work exhibits the following clinical value and limitations. First, we constructed a novel cuproptosis-related lncRNA prognostic model and validated for the first time a novel pathway of lncRNA regulation of cuproptosis for OSCC. Second, the model can be applied to be an independent predictor of OSCC patients and can be adopted for the identification of the prognosis and immune response of OSCC patients, providing a novel idea to guide the immunotherapy of OSCC. Third, we also predicted drug sensitivity, which provides a new direction to promote future precise and personalized targeted therapy. The next research plan of this project is to collect more samples, use clinical follow-up data to demonstrate the value of our prognostic model, and then conduct in vitro and in vivo experiments further to investigate the potential mechanism of 8-CRL in OSCC.

## Conclusion

The 8-CRL prognostic model, developed for predicting prognosis, immune response, and drug sensitivity in oral squamous cell carcinoma (OSCC), should be given significant attention in the construction of novel CRL models. Further exploration of this model could prove instrumental in developing effective models for OSCC.

## Materials and methods

### The collection of data

The Cancer Genome Atlas (TCGA) database (https://portal.gdc.cancer.gov/repository) provided transcriptome profiles and clinical characteristics of 373 OSCC samples, containing 32 normal and 341 tumor samples.

### Identification of cuproptosis-related LncRNAs

To identify the CRLs, a total of 19 cuproptosis-related genes (CRGs) were summarized from recently published literature [6] (Supplementary Table S2). Using the GENCODE website (https://www.gencodegenes.org/, as of September 3, 2021) to retrieve signature GTF annotation files, 16,876 lncRNA were identified from the TCGA-seq OSCC RNA data to distinguish the mRNA and lncRNA. This study used Pearson’s correlation analysis which was considered an accepted method to investigate the correlation between coding genes and lncRNAs to examine the co-expression of cuproptosis-related genes (CRGs) in each signature among OSCC samples. We have set various R values based on the published documents and chosen *R* > 0.4 and *P* < 0.001 as the best cutoff value eventually. Sankey plots were made by the “ggalluvial” R package (version of 4.1.0; https://www.r-project) to demonstrate the degree of association between CRL and CRG. With the aim of examining the prognostic ability of these CRLs, OSCC samples were classified into training and test group in a random and equal manner. For the purpose of screening lncRNAs in relationship to prognosis, Univariate Cox regression analysis was adopted. In addition, LASSO regression analysis was used to screen for lncRNAs that were significantly associated with overall survival (OS) in OSCC patients. This study conducted a multifactorial Cox regression analysis to generate the best model. Finally, Eight lncRNAs associated with cuproptosis were considered as prognostic factors. Risk scores were calculated with the following equations: $\text{R}\text{i}\text{s}\text{k} \text{s}\text{c}\text{o}\text{r}\text{e}={\sum }_{\text{i}= 1}^{\text{n}}\text{C}\text{o}\text{e}\text{f}\left(\text{i}\right)\times \text{x}\left(\text{i}\right)$, x(i) and Coef(i) represent the expression levels and corresponding coefficients of each prognostic lncRNA, respectively.

### Construction of the cuproptosis-related prognostic model

We adopted the model formula for calculating scores for the samples. With the median risk score of the training group, there were high-risk and low-risk groups. R packages (survival and survminer) were applied to Kaplan-Meier survival analysis, risk score curve, and survival status scatter plot to the training group to contrast the survival difference and confirmed in the testing and total cohorts. With the risk scores and survival statistics, the risk heatmap of the model lncRNAs was plotted using the “pheatmap” package. Clinical subgroups were established on the basis of age, sex, clinical stage, and pathological grade. Kaplan-Meier survival analysis was adopted for verifying whether the model can be employed for individuals exhibiting various clinical features.

### Validation of the prognostic model

For the purpose of evaluating the feasibility of the model, prognostic features were assessed in the testing and entire cohorts. We made univariate and multivariate regression analyses to demonstrate whether the prognostic model can determine the prognosis of OSCC patients independently of other clinical factors. Additionally, with the use of the “timeROC” package, the 1, 3, and 5 years receiver operating characteristic (ROC) curves were made, aiming to validate further the predictive ability of the established risk prognostic model. Based on the R packages “rms” and “regplot”, this study built a Nomogram using the 8-CRL signature, including risk scores, age, and stage information. Using the nomogram, this study assessed the prognosis of OSCC patients at 1, 3, and 5 years. Calibration curves were used for identifying the accuracy and reliability of the nomogram. One patient was chosen randomly to demonstrate the nomogram’s predictive utility. To study the distribution of high- and low-risk populations, we adopted principal component analysis (PCA).

### Functional and pathway enrichment analysis

Cuproptosis-related prognostic model was created through TCGA data analysis, and patients were divided into high-risk group and low group. The “limma” package was first used to detect differentially expressed genes (DEGs) between high-risk and low-risk groups. Genes which had |logFC|> 1 and FDR < 0.05 were of statistical significance. In addition, in order to identify the effects of the two risk groups on cell function and the different pathways of enrichment, we used gene ontology (GO) pathway enrichment analysis and Kyoto Encyclopedia of Genes and Genomes (KEGG) pathway enrichment analysis.

### Immune function and Tumor Immune Dysfunction and Exclusion (TIDE) score

Using the CRL model, we compared single-sample gene set enrichment analysis (ssGSEA) and the “gsea” package, aiming to identify the differences in immune function. Besides, a heatmap was applied to display the differences in immune function. Additionally, the TIDE score was adopted to identify the outcome and response of cancer patients to immunotherapy. TIDE scores, CAF, IFNG, CD8, CD274, MDSC, T-cell dysfunction, Merck18, TAM M2, and T-cell exclusion scores were acquired from the TIDE website (http://tide.dfci.harvard.edu).

### Tumor Mutation Burden (TMB) analysis

A waterfall plot was made with the “maftools” package, aiming to evaluate and compare the frequency of gene mutations. Then, the “limma” and “ggpubr” packages were adopted for comparing the survival analysis and Tumor Mutation Burden (TMB) between different subgroups as well as to compare the prognosis and tumor mutation status.

### Drug sensitivity analysis

“pRophetic” program package was used to evaluate the median inhibitory concentration (IC50) of 138 common antitumor agents in order to predict the response to chemotherapy agents in patients with OSCC in two different risk subgroups. The IC50 values between the two risk groups were analyzed by the Wilcoxon signed-rank test.

### Real-time quantitative PCR analysis to confirm the expression of CRL

Human normal oral keratinocytes (HOK), human oral squamous cell line (CAL-27), and human tongue squamous cell carcinoma cells (SCC25) were purchased from ScienCell. All the cells were cultivated in high-glucose DMEM medium covering 10% fetal bovine serum as well as 1% penicillin/streptomycin solution. The extraction of total RNA was performed from the cells with TRIzol. Subsequently, a reverse transcription kit was employed to synthesize cDNA. qRT-PCR was made with 2X SG Fast qPCR Master Mix and a Thermo Fisher QuantStudioTM1 Plus fluorescent quantitative PCR instrument. Through the 2-ΔΔCT method, the levels of each gene were quantified with GAPDH being the internal reference for normalization. We can find primer sequences in Supplementary Table S3 (The primer sequence number of AL132800.1 was not observed in the literature and databases, so it cannot be confirmed at this time).

### Statistical analysis

Statistical analyses and visualization were mainly conducted by R software (version 4.1.0). Student’s t-test, one-way ANOVA and Welch ANOVA were used to calculate differences between two groups or more. A log-rank test with the best cutoff value was used to plot a Kaplan-Meier (KM) survival curve to analyze OS. Univariate, Lasso, and multivariate Cox regression analyses were established to evaluate the prognostic significance. Pearson correlation analysis was used to get the correlation of gene expression. ROC and its AUC curve were adopted to estimate the reliability and sensitivity of the prognostic signature. Two-sided *p* < 0.05 was regarded as statistically significant.

### Supplementary Information

**Additional file 1:** **Table S1.** 139 differentially expressed genes (DEGs) between high-risk and low-risk groups.

** Additional file 2: Table S2. **19 cuproptosis-related genes (CRGs).

** Additional file 3: Table S3. **Primer sequence of genes in qRT-PCR.

## Data Availability

The original contributions are contained in the article/Supplementary Material. Besides, further inquiries can be made to the corresponding authors. The RNA-seq data sets and patient clinical characteristics were downloaded from the TCGA database (https://ocg.cancer.gov/programs/TCGA).

## Abbreviations

AUC: Areas under the curve
BP: Biological processes
CAF: Cancer-associated fibroblast
CC: Concerning cellular
CD: Cluster of differentiation
CRG: Cuproptosis-related gene
CRL: Cuproptosis-related lncRNA
DEG: Differentially expressed gene
GO: Gene Ontology
HNSCC: Head and neck squamous cell carcinoma
HOK: Human normal oral keratinocyte cells
IC50: Half-maximal inhibitory concentration
IFNG: Interferon-gamma
KEGG: Kyoto Encyclopedia of Genes and Genomes
K–M: Kaplan–Meier
LASSO: Least absolute shrinkage and selection operator
LncRNA: Long non-coding RNA
MDSC: Myeloid-derived suppressor cell
MF: Molecular function
OS: Overall survival
OSCC: Oral squamous cell carcinoma
PCA: Principal coordinate analysis
qRT-PCR: Quantitative reverse transcription–polymerase chain reaction
RCD: Regulated cell death
ROC: Receiving operating characteristic
ssGSEA: Single-sample gene set enrichment analysis
TAM: Tumor-associated macrophages
TCGA: The Cancer Genome Atlas
TIDE: Tumor immune dysfunction and exclusion
TMB: Tumor Mutation Burden
