# Supplementary material for: Bioinformatic analysis and experimental validation of cuproptosis-related LncRNA as a novel biomarker for prognosis and immunotherapy of oral squamous cell carcinoma
Source: Hereditas. 2024 Feb 27;161:10. doi: 10.1186/s41065-024-00311-5 (PMC10898041; doi:10.1186/s41065-024-00311-5)
Supplement: Supplementary file 1 — Additional file 1: Table S1. 139 differentially expressed genes (DEGs) between high-risk and low-risk groups. [file 41065_2024_311_MOESM1_ESM.docx]

**Supplementary Table S1.** 139 differentially expressed genes (DEGs) between high-risk and low-risk groups

| GLB1L2 | MAL | FOXL2 | NPM1P21 | GJB7 | GALR2 |
| --- | --- | --- | --- | --- | --- |
| FGF19 | CASC8 | MROH2A | DEFB126 | HAL | PDE6B |
| TRBJ1-4 | HOXA11 | C8G | AC083841.1 | KREMEN2 | TNNI3 |
| CLDN17 | FAM71E1 | AC023310.4 | UCHL1 | HOXA9 | GAL |
| PCAT19 | AL513318.1 | PRAME | STIM2-AS1 | CAPS | SSC4D |
| SPINK1 | LCE1B | RPS26P47 | AC025244.1 | POU6F2-AS2 | KRT1 |
| PCOLCE2 | HCG22 | TRBJ1-6 | CRNN | ARG1 | CDIPTOSP |
| CD70 | CLDN6 | LCE2B | AC011632.1 | KRT8P26 | FLG |
| DSG1 | KPRP | NEU2 | AC083967.1 | DLL3 | LCEP3 |
| KRT3 | AC245100.3 | B4GALNT4 | SLC5A1 | KRT2 | HHIPL2 |
| GCOM1 | KRT78 | LINC01980 | NKX2-5 | C1QTNF12 | CLDN3 |
| ACER1 | SCGB3A1 | TFPI2 | AC025575.2 | IGHV7-81 | PPIAP31 |
| SLURP1 | ENDOU | GSDMA | PDIA2 | NPW | AP005233.2 |
| SMIM1 | ZNF229 | HAGHL | SOX10 | LCE6A | CEACAM7 |
| NPM2 | FSD1 | TPT1P5 | FAM25BP | PLA2G4D | LCE3E |
| IGKV2-28 | SOHLH1 | ASPRV1 | AC091133.5 | DNAJC22 | AC007182.2 |
| FGFBP2 | PCSK1N | ADRA2C | TRBJ1-3 | FLG2 | TMEM121 |
| ZIC1 | RPL39L | LCE1A | HOXB9 | RBP4 | BARX1 |
| RNF222 | DPYSL4 | PCYT1B | CGREF1 | KRTAP19-1 | LCE2A |
| ALX1 | LCE2C | LINC01807 | RHPN1 | LCE3A | GPR27 |
| GDF15 | SPINK7 | BPIFA1 | GSTM2 | KRT6C | CYP26A1 |
| RETN | WFDC12 | LCE2D | TRPM2-AS | MLXIPL | CEL |
| BEX1 | HPN | AL138916.1 | BOK-AS1 | SLC35G1 | COL9A3 |
| TCF15 |  |  |  |  |  |
