# Supplementary material for: Bioinformatic analysis and experimental validation of cuproptosis-related LncRNA as a novel biomarker for prognosis and immunotherapy of oral squamous cell carcinoma
Source: Hereditas. 2024 Feb 27;161:10. doi: 10.1186/s41065-024-00311-5 (PMC10898041; doi:10.1186/s41065-024-00311-5)
Supplement: Supplementary file 2 — Additional file 2: Table S2. 19 cuproptosis-related genes (CRGs). [file 41065_2024_311_MOESM2_ESM.docx]

**Supplementary Table S1.** 19 cuproptosis-related genes (CRGs)

| **Genes** | **Description** |
| --- | --- |
| ATP7A | copper transport ATPase α |
| ATP7B | copper transport ATPase β |
| CDKN2A | cyclin- dependent kinase inhibitor 2A |
| DBT | dihydrolipoamide branched chain transacylase E2 |
| DLAT | drolipoamide S- acetyltransferase |
| DLD | dihydrolipoamide dehydrogenase |
| DLST | dihydrolipoamide S-Succinyltransferase |
| FDX1 | ferredoxin 1 |
| GCSH | glycine cleavage system protein H |
| GLS | glutaminase |
| LIAS | lipoic acid synthetase |
| LIPT1 | lipoyl transferase 1 |
| LIPT2 | lipoyl transferase 2 |
| MTF1 | metal-regulatory transcription factor-1 |
| NFE2L2 | nuclear factor (erythroid derived 2)-like 2 |
| NLRP3 | nucleotide-binding oligomerization domain-like receptor protein 3 |
| PDHA1 | pyruvate dehydrogenase E1 subunit alpha 1 |
| PDHB | pyruvate dehydrogenase E1 subunit beta |
| SLC31A1 | solute carrier family 31 member 1 |
