# Supplementary material for: Bioinformatic analysis and experimental validation of cuproptosis-related LncRNA as a novel biomarker for prognosis and immunotherapy of oral squamous cell carcinoma
Source: Hereditas. 2024 Feb 27;161:10. doi: 10.1186/s41065-024-00311-5 (PMC10898041; doi:10.1186/s41065-024-00311-5)
Supplement: Supplementary file 3 — Additional file 3: Table S3. Primer sequence of genes in qRT-PCR. [file 41065_2024_311_MOESM3_ESM.docx]

**Supplementary Table S2.** Primer sequence of genes in qRT-PCR

|  | Forward sequence | Reverse sequence |
| --- | --- | --- |
| THAP9-AS1 | CGCCATCGTCCTTCTTGTGA | GCTTCCCCATTATCTCCGCA |
| WDFY3-AS2 | GAAACGCAAAGGCTACTAGAC | AGTTTCTTTCCATCTGGTCCT |
| LINC00847 | AACGCTGCCTCTGTGGAAGTCTC | CGCTCTGCTCTCCCGCCATC |
| GCC2-AS1 | GGCTCTGTGGTAAGGCTCTG | ACTGACGAATAACTTGGGGCA |
| CDKN2A-DT | CAGCGTGGACAGGAGCATCT | TGTGAGGTTGCGAATGACTGC |
| STARD4-AS1 | GCCTGCCCTGTCAAAAGAAG | GTGCTTCTGCTGTCTGGTTG |
| AC005746.1 | GCGAGCCAACGACATCCTGAAG | GTAGGCAGTGACGGCGATGAAG |
| GAPDH | ACAACTTTGGTATCGTGGAAGG | GCCATCACGCCACAGTTTC |
